# Transcriptome profiles associated with selenium-deficiency-dependent oxidative stress identify potential diagnostic and therapeutic targets in liver cancer cells

**DOI:** 10.3906/biy-2009-56

**Published:** 2021-04-20

**Authors:** Damla GÖZEN, Deniz Cansen KAHRAMAN*, Kübra NARCI, Huma SHEHWANA, Özlen KONU, Rengül ÇETİN-ATALAY

**Affiliations:** 1 Cancer Systems Biology Laboratory, Department of Health Informatics, Graduate School of Informatics, Middle East Technical University, Ankara Turkey; 2 Department of Biological Sciences, National University of Medical Sciences, Rawalpindi Pakistan; 3 Department of Molecular Biology and Genetics, Faculty of Science, Bilkent University, Ankara Turkey

**Keywords:** Hepatocellular carcinoma, selenium, oxidative stress, transcriptome-wide analysis

## Abstract

Hepatocellular carcinoma (HCC) is one of the most common cancer types with high mortality rates and displays increased resistance to various stress conditions such as oxidative stress. Conventional therapies have low efficacies due to resistance and off-target effects in HCC. Here we aimed to analyze oxidative stress-related gene expression profiles of HCC cells and identify genes that could be crucial for novel diagnostic and therapeutic strategies. To identify important genes that cause resistance to reactive oxygen species (ROS), a model of oxidative stress upon selenium (Se) deficiency was utilized. The results of transcriptome-wide gene expression data were analyzed in which the differentially expressed genes (DEGs) were identified between HCC cell lines that are either resistant or sensitive to Se-deficiency-dependent oxidative stress. These DEGs were further investigated for their importance in oxidative stress resistance by network analysis methods, and 27 genes were defined to have key roles; 16 of which were previously shown to have impact on liver cancer patient survival. These genes might have Se-deficiency-dependent roles in hepatocarcinogenesis and could be further exploited for their potentials as novel targets for diagnostic and therapeutic approaches.

## 1. Introduction

Cancer is one of the major public health problems worldwide, being the second most deadly disease, according to the World Health Organization, and nearly 30 million new cases are estimated to occur globally by 2040 (Siegel et al., 2020). Cancer therapeutics has shifted away from the conventional chemotherapeutic drugs towards targeted therapeutic strategies to provide higher efficacy with lower toxicity. For finding novel genes for diagnosis and targeted therapy, it is crucial to determine cancer cell characteristics in more detail and provide more insight into molecular mechanisms behind carcinogenesis. 

Hepatocellular carcinoma (HCC) is the fourth most fatal and the fifth most frequent cancer worldwide (Bray et al., 2018). Molecular mechanisms involved in HCC are more complex than other cancers. One of the apparent characteristics of liver cancer cells is their increased resistance to various stress conditions such as chronic viral infections or toxins. Therefore, it is essential to determine the stress response gene expression profiles of these cells due to their involvement in hepatocarcinogenesis (Di Maso et al., 2015).

Generally, cells respond to stress in a variety of ways, from activation of pathways that promote survival to initiating programmed cell death to eliminate damaged cells (Fulda et al., 2010). One of the stress factors eukaryotic cells try to adapt to is oxidative stress conditions for which cells have evolved different responses. Oxidative stress results from the inability of the biological system to detoxify the reactive intermediates or to compensate the resulting damage that is formed as a result of systemic accumulation of reactive oxygen species (ROS). Disturbances in the normal redox state of cells produce peroxides and free radicals that damage all components of the cell, causing toxic effects. It may also suppress apoptosis and promote proliferation, invasiveness, and metastasis, leading to carcinogenesis (Halliwell et al., 2007). 

Selenium (Se) is a trace element which is required for human health, and its deficiency results in various abnormalities. It is found in the structure of selenocysteine (SeCys) amino acid, which is the building block of selenoproteins. There are about 25 different selenoproteins identified in humans with various functions, including antioxidant and redox signalling functions (Papp et al., 2007). Normally, deficiency of Se results in oxidative stress, leading to apoptosis. However, in a previous study we performed with HCC cell lines, it was found that 10 of 13 HCC cell lines tolerated Se deficiency to escape apoptosis, and most of these tolerant cell lines had Hepatitis B virus (HBV) sequence integrated into their genome, indicating that this virus might have a role in that acquired tolerance (Irmak et al., 2003). This study was repeated with two isogenic HCC cell lines; HepG2 and HepG2-2.2.15 cells, to test their response to Se deficiency. These two cell lines have the same genome, except the HBV genomic integration in HepG2-2.2.15 cells, and it was found that HepG2 cells were Se-deficiency-sensitive while HepG2-2.2.15 cells tolerated the absence of Se to survive. Although the underlying mechanism remains unclear, it could be a result of both intrinsic and/or acquired mechanisms. 

During the viral genome integration into HepG2-2.2.15 cells, an increase in ROS generation is expected in the cells, which alters the cellular gene expression (Waris et al., 2005). This would result in an intrinsic variation in the gene expression of HepG2-2.2.15 cells compared to HepG2 cells, independent of the Se status of the growth medium. In addition to this intrinsic effect, an acquired mechanism might be at work due to the absence of Se. Understanding the molecular mechanism behind the Se tolerance might give valuable information about cellular response mechanisms gained by some cancer cells to escape from oxidative-stress-dependent apoptosis. 

Large-scale expression analysis using microarray or RNAseq data has great potential to enlighten the changes that occur at the molecular level resulting in hepatocellular carcinogenesis (Chen et al., 2020). In this study, systems-based approaches were used to identify genes important in oxidative stress resistance mechanisms, which could be further exploited as novel drug targets or for diagnostic purposes in HCC.

## 2. Materials and methods

### 2.1. Cell lines and microarray experiment

As we described in our previous study (Irmak et al., 2003), oxidative-stress-resistant HepG2-2.2.15 cell line (HT (Head-to-Tail)-HBV integration) and -sensitive HepG2 cell line were grown in DMEM (Dulbecco’s Modified Eagle’s medium) (2 × 105 cells in 10 mm culture dish) (Gibco, Thermo Fisher Scientific, MA, USA) with 0.01% FCS (BioChrom, Berlin, Germany) either supplemented with 0.1 μM Na2SeO3 (Sigma, Taufkircher, Germany) (Se positive) or not (Se negative) for 3 days in duplicates at 37 °C in an incubator containing 5% CO2. They were harvested on the 1st, 2nd, and 3rd days of the treatment, and RNA isolation was performed. For each day, gene expression data were acquired by Human Genome U133 Plus 2.0 Array Affymetrix Array. The data discussed in this publication have been deposited in NCBI’s Gene Expression Omnibus (Edgar et al., 2002) and are accessible through GEO Series accession number GSE163950https://www.ncbi.nlm.nih.gov/geo/query/acc.cgi?acc=GSE163950.

### 2.2. Preprocessing of microarray data

The qualities of arrays were checked using simpleaffy package available in RMiller CJ (2020). simpleaffy: Very simple high level analysis of Affymetrix data [online]. https://bioconductor.org/packages/simpleaffy/ accessed on 5.04.2020. Robust multiarray average (RMA) background correction and quantile normalization of the data were performed.

### 2.3. Determination of differentially expressed genes (DEG)s

To identify DEGs between HepG2 and HepG2-2.2.15 cells in the presence or absence of Se, limma package in R was used (Ritchie et al., 2015). The design matrix was organized so that it involved both cell lines (HepG2 or HepG2-2.2.15) and Se status (present or absent) information, separately for each day to fit the data to a linear model in the first step.

In line with the design matrix, the contrast matrix was designed in a specific way to determine the effects of cell line and Se status on gene expression separately for each day. 

The first two comparisons were based on the identification of the genes that were differentially expressed between HepG2 and HepG2-2.2.15 cells in the presence of Se (Se+ HepG2 vs. Se+ HepG2-2.2.15) or absence of Se (Se–HepG2 vs. Se–HepG2-2.2.15) and named “between cell line comparisons”. The last two comparisons aimed to answer the question of which gene expressions were altered within a cell line in the presence or absence of Se for HepG2 (Se–HepG2 vs. Se+ HepG2) or HepG2-2.2.15 (Se–HepG2-2.2.15 vs. Se+ HepG2-2.2.15) and called “within cell line” comparisons. The p-value threshold was taken as 0.01 while selecting the DEG lists. 

### 2.4. Clustering analysis of differentially expressed genes 

Z-score of each gene in the defined DEG list was calculated for clustering of the samples (Kreyszig, 1979). Heatmaply package (Galili et al., 2017) in R-Bioconductor was used to draw the heat maps. 

### 2.5. Gene set enrichment analysis with DEGs

A score called ‘DEG score’ was calculated (Cavga et al., 2019), providing the normalization of the results for each DEG relative to each other. DEG scores were calculated by subtracting log2 fold change values of HepG2 DEGs in within cell line comparison results from that of HepG2-2.2.15 DEGs and taking the absolute values for each. Gene set enrichment analysis (GSEA) (Subramanian et al., 2005) was performed using DEG lists and for each gene, preranked inputs were determined using DEG score values. The Molecular Signatures Database (MSigDB) hallmark or GO-Biological Process data collection was used to search for enriched gene sets using the default “weighted” enrichment statistic parameter and “meandiv” normalization. Enriched gene sets with FDR q-values < 0.25 were considered significant. 

### 2.6. Network analysis

#### 2.6.1. STRING 

In this study, the identified DEG lists were further analyzed to gain more insight into their biological mechanisms by obtaining experimentally validated protein–protein interaction (PPI) information from STRING database (Jensen et al., 2009). Hub proteins with high degree or betweenness centrality values were further analyzed. 

#### 2.6.2. Prize-collecting Steiner tree

Prize-collecting Steiner tree (PCST) (Tuncbag et al., 2016) approach was used to identify the interactions through the DEG lists by using the information provided by human interactome data. OmicsIntegrator software’s Forest module was used to determine the subnetwork in the set of DEGs. 

To construct meaningful trees, β, ω, and µ input parameters must be chosen correctly, and for each DEG list, the optimum values of those input parameters were determined separately by the Forest-tuner algorithmhttps://github.com/gungorbudak/forest-tuner accessed on 25.06.2020 to generate trees with higher number of prices and smallest mean degrees of nodes. 

In our analysis, STRING protein–protein interaction database v10 was used to extract the interactome reference set (Jensen et al., 2009). In this database, each edge is given a confidence score between 0 and 1 according to the reliability of the data source. The edges that have confidence scores higher than 0.7 were chosen. These scores were used to determine the costs by OmicsIntegrator.

### 2.7. Survival data

Kaplan–Meier survival analysis was performed by generating plots for each gene of interest by KM plotterMenyhart et al. (2018). Kaplan-Meier Plotter [online]. Website https://kmplot.com, which plots the survival data of liver cancer patients according to liver cancer RNA-seq results from TCGA including all pathology and patient types. The results with logrank p-values smaller than 0.05 were accepted to be significant. 

## 3. Results

### 3.1. Identification of differentially expressed selenium deficiency resistance and sensitivity genes

**Figure 1:**
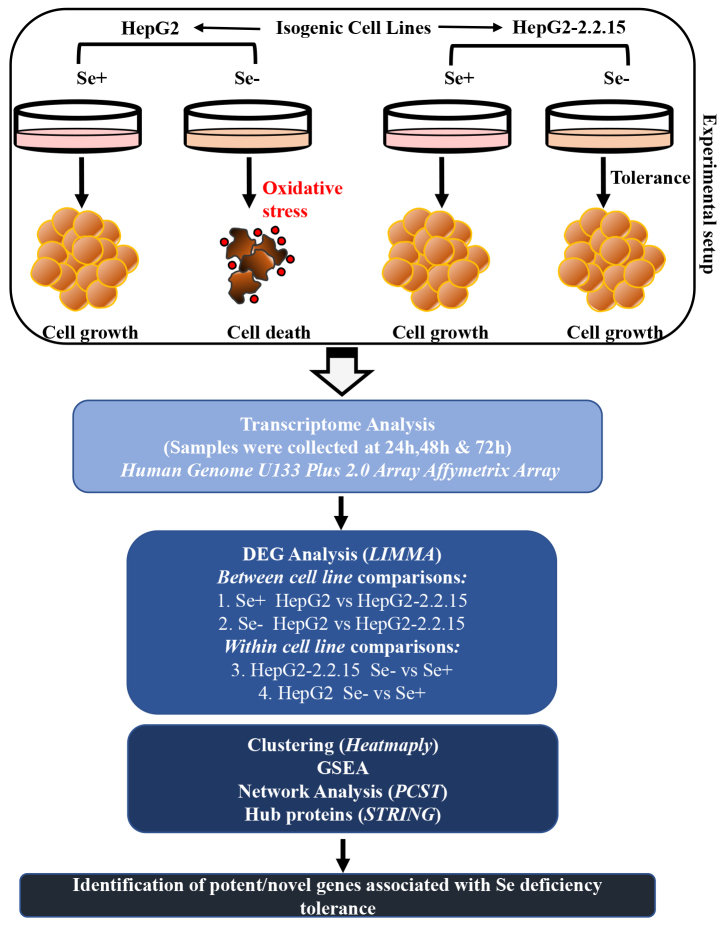
Illustration of the experimental design used to generate and analyze the data. HepG2 and HepG2-2.2.15 cells (two isogenic HCC lines with the difference of HBV genome integration in HepG2-2.2.15 cells) were grown in the presence or absence of Se to perform transcriptome analysis. The results were examined by further bioinformatics methods to determine the differential response mechanisms.

Transcriptome-wide gene expression analysis was performed in two different cell lines with or without selenium treatment in a time-dependent manner (three time-points; day 1, 2, and 3), and the workflow of the analysis, which was followed throughout the study, is summarized in Figure 1.

To analyze the results and determine DEGs that are important for the tolerance to Se-deficiency-dependent oxidative stress, limma package in R was used. As explained in methods, four comparisons were performed separately for day 1, 2, and 3.

**Figure 2:**
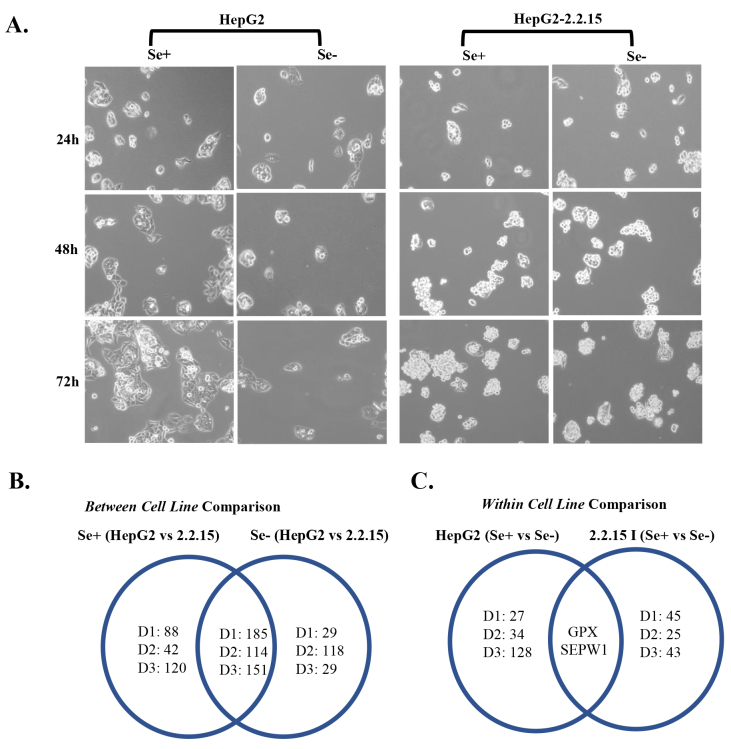
Experimental groups and differential expression analysis of HepG2 and HepG2-2.2.15 cell lines. (a) Representative live images of HepG2 and HepG2-2.2.15 cells grown in Se+ or Se– media for 72 h. The DEG numbers were identified as a result of the (b) between cell line and (c) within cell line comparisons by limma analysis. Venn diagrams were drawn in order to indicate the common and unique DEGs within indicated comparison groups for each day. 2.2.15: HepG2-2.2.15, D1: 24 h, D2: 48 h; D3: 72 h.

Representative live images of HepG2 and HepG2-2.2.15 cells grown in Se+ or Se– media for 72 h showed that the effects of Se deficiency on cell confluency of HepG2 cells was detected most significantly on the 3rd day when compared to those incubated in Se+ media (Figure 2a). Meanwhile, HepG2-2.2.15 cells were able to grow under both conditions. These results were in parallel with our previous findings (Irmak et al., 2003), where HepG2 cells underwent apoptotic cell death under selenium deficiency, while HepG2 2.2.15 cells displayed tolerance and similar growth rates under Se– and Se+ culture conditions. 

Numbers of DEGs for each condition were identified per comparison respectively as indicated in Figures 2b and 2c. By the first two limma analyses between cell line comparisons (Figure 2b), the DEGs that were only differentially expressed in Se– HepG2 vs. HepG2-2.2.15 and not in Se+ HepG2 vs. HepG2-2.2.15 were further named ‘Se-deficiency-dependent effect genes’ since they were altered depending on the absence of Se. The shared DEGs in both Se+ and Se– comparisons were named ‘HBV-integration effect genes’ in this study (Figure 2b), as their expression differences were independent of the Se status; which is thought to be about the integration of HBV viral genome. By the last two limma analyses within each cell line comparisons (Figure 2c), the expression of GPX and SEPW1, the two selenoproteins, decreased in the absence of Se in both cell lines. This could be expected due to the direct dependence of their expression on the presence of Se. The DEGs that were not shared in common were named ‘Se-deficiency-dependent effect genes’ and further examined since they were directly related to the different reactions each cell line gave to the deficiency of Se.

### 3.2. Differentially expressed gene clustering analysis of the between cell line comparisons 

**Figure 3:**
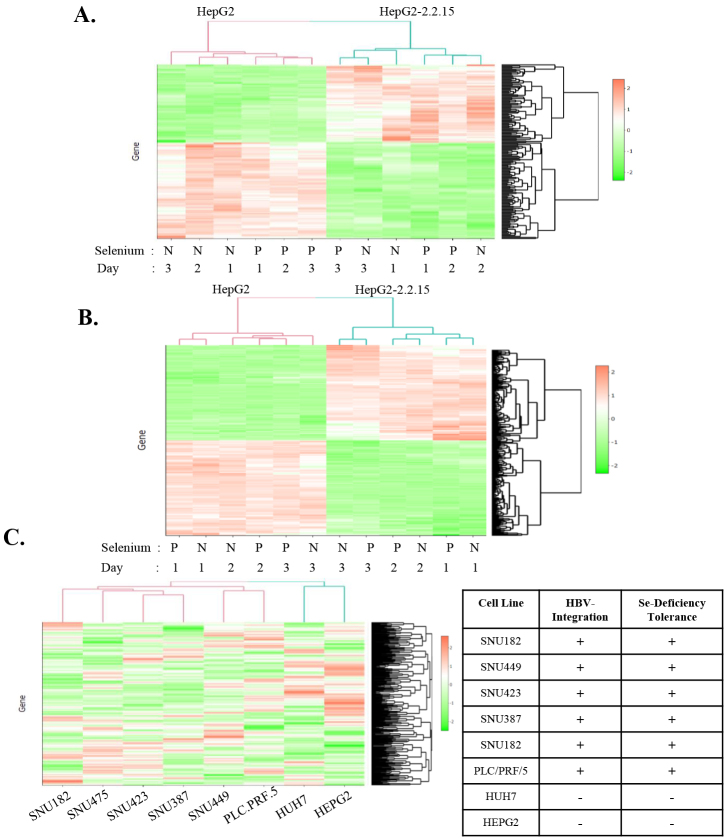
The heat map drawn with (a) Se-deficiency effect genes and (b) HBV-integration effect genes. (c) The heat map drawn with Z scores of HBV-integration effect genes calculated for the expression levels of 8 different cell lines taken from CCLE results; HBV-integration and Se-deficiency tolerance conditions of cell lines were depicted in the table.

For between cell line comparisons, the z-scores for expressions of the Se-deficiency-dependent effect genes in each sample were calculated and used to draw a heat map clustering samples all together for day 1 (D1), day 2 (D2), and day 3(D3) data (Figure 3a). As expected, HepG2 and HepG2-2.2.15 cells were clustered in two distinct groups. PPAP2A, HOXD1, and CLYBL genes were found to be the most DEGs between the two cell lines. A similar clustering was performed for the HBV-integration effect genes. HepG2 and HepG2-2.2.15 cells were clustered separately again (Figure 3b).

FGF13, GPC3, and MAP7D2 genes were identified as the DEGs with the smallest p-values between HepG2 and HepG2-2.2.15. In parallel, we also used the HBV-integration effect gene lists to draw heat maps for the comparative expression levels of these DEGs in 8 different hepatocellular cancer cell lines taken from Cancer cell line encyclopedia databasehttps://portals.broadinstitute.org/ccle. Six of these cell lines have HBV genomic integration and were found to be resistant to Se-deficiency-dependent oxidative stress (SNU182, SNU475, SNU423, SNU387, SNU449, and PLC.PRF.5) in a previous study (Irmak et al., 2003) while two of them (HepG2 and HUH7) are known to be virus-free and sensitive to Se-deficiency-dependent oxidative stress. Dendrograms showed clustering of the two Se-deficiency sensitive cell lines together; distinctly from the other 6 cell lines, further supporting the HBV-integration effect hypothesis stating that differential expression of these genes was indeed related to the HBV genome integration independent of the cell’s Se status (Figure 3c).

### 3.3. Gene set enrichment analysis of isogenic HepG2 and HepG2-2.2.15 cells 

**Figure 4:**
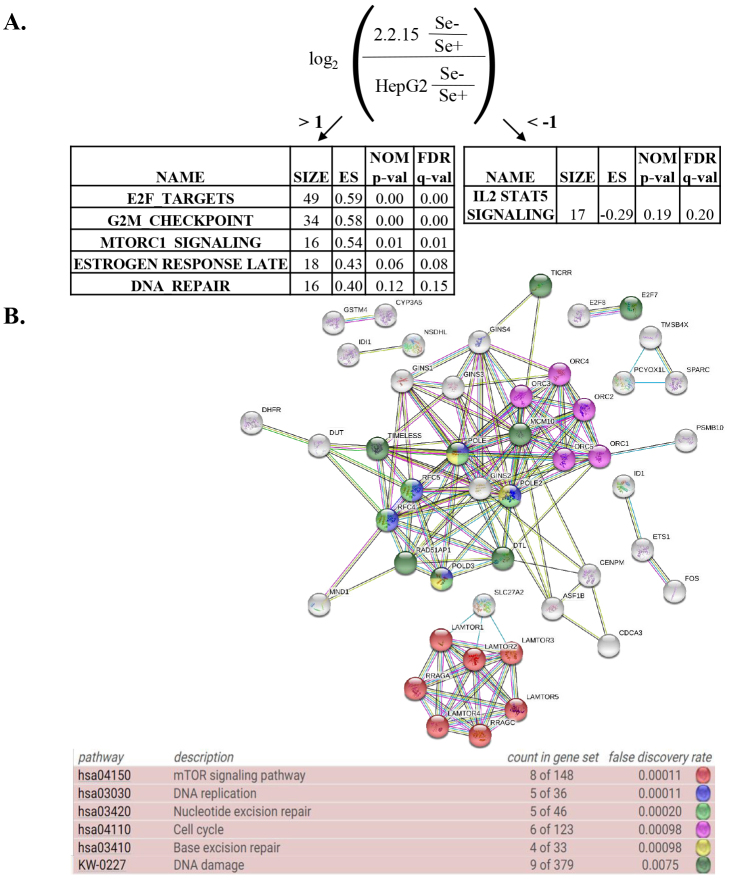
Enrichment results of within cell line comparison DEG list (a) DEG scores were given for each gene for Day 3 according to the indicated formula and the GSEA was performed to find the enriched Hallmark pathways with their enrichment scores, (b) the STRING was used to perform pathway analysis and to find the enriched pathways. Only top 5 KEGG pathways with the smallest FDR values, and Uniprot annotated keyword related to damage was shown for clarity. ES: enrichment score, NOM p-val: nominal p value, FDR: false discovery rate.

For the within cell line comparison results, DEG scores were calculated (Cavga et al., 2019) as explained in methods (Figure 4a). The pathways enriched in lists with positive DEG scores (DEG scores > 1) indicate that the relevant genes were relatively upregulated in HepG2-2.2.15 when normalized to HepG2 cells within Se– vs. Se+ comparisons. Accordingly, the activities of these pathways were higher in HepG2-2.2.15 cells in response to Se- deficiency. In contrast, the pathways enriched in lists with negative DEG scores (DEG scores < 1) indicated a lower activity in HepG2-2.2.15 cells in response to Se-deficiency compared to HepG2. GSEA results have revealed that the relatively upregulated genes in HepG2-2.2.15 cells were found to be more related to DNA-repair, G2M checkpoint, oxidation reduction, and MTORC1 signaling pathways, which might be key pathways for the acquired tolerance gained by HepG2-2.2.15 cells to Se-deficiency-dependent oxidative stress as they all seem to have linked to survival mechanisms. The enriched pathways in the DEG lists with the DEG scores higher than 0 were found to be related to stress response, repair mechanisms, and cell cycle (Table S1). On the contrary, the relatively downregulated genes in HepG2-2.2.15, when normalized to those in HepG2 were found to be enriched in IL2-STAT5 signaling pathway, which is known to have functions related to apoptosis (Zamorana et al., 1998) and might indicate lower apoptotic phenotype in within HepG2-2.2.15 comparison relative to that of HepG2. The same DEG lists were further used to generate a network of predicted associations between proteins of interest by STRING, and enriched pathways found on this network were consistent with GSEA results (Figure 4b).

### 3.4. Pathway analysis to identify key genes related to selenium-dependent oxidative stress tolerance 

**Figure 5:**
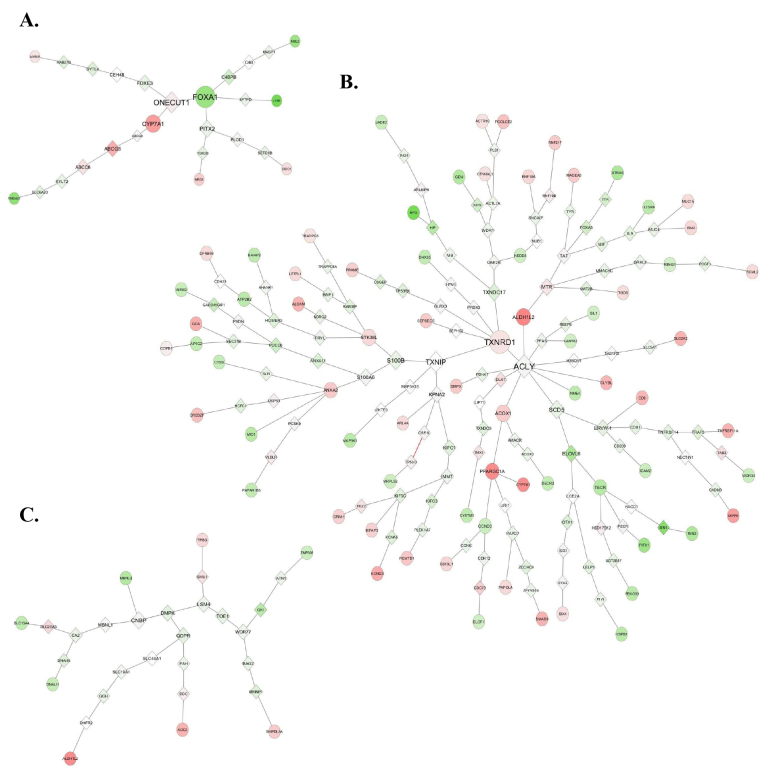
The trees constructed by PCST algorithm for acquired HepG2 vs. HepG2-2.2.15 DEG lists at day 1 (a), day 2, (b) and day 3 (c). STRING database was used as the reference database. Circles indicate the terminal nodes and diamonds indicate the Steiner nodes. Node colors indicate expression level difference of each gene between cell lines, green and red indicating negative and positive fold changes, respectively.

PCST algorithm was used to construct trees for Se– deficiency effect genes by taking STRING as the reference dataset (Figure 5). PCST constructs optimum trees from the given DEGs (terminal nodes) by using human interactome data as a reference to find the shortest path between the nodes. The Steiner nodes were determined by the algorithm (shown by diamond in the figures) and the nodes that have high betweenness centrality values were identified in order to investigate in more detail (biggest nodes in each tree) since they might play some key roles considering their connecting positions between different branches of the tree. 

Most of the genes with high betweenness centrality (both DEGs and Steiner nodes) were found to have roles related to oxidative stress response either with impacts in oxidative-stress-dependent apoptosis or antioxidant pathways (Figure 5). In day 1 results, FOXA1, an oxidoreductase which has a proapoptotic role, and CYP7A1 were differentially expressed while ONECUT1 with roles in cell cycle regulation and PITX2 involved in oxidative stress response were detected as Steiner nodes, i.e. differentially expressed but might be critical in the differential response (Figure 5a). In day 2 results, the DEGs TXNRD1 and ALDH1L2 and the Steiner nodes ACLY, TXNIP, SCD5, MTR, and TXNDC17 all exhibiting oxidative stress and redox homeostasis-related functions (Figure 5b) emerged. In day 3 results, the Steiner nodes with high betweenness centrality values included LSM4 that is associated stress response and CNBP, DMPK, QDPR with antioxidant functions (Figure 5c). This has supported the idea that these DEGS could be the key elements in the acquired tolerance gained by HepG2-2.2.15 cells to Se-deficiency-dependent oxidative stress.

### 3.5. Definition and clinical significance of selected genes related with oxidative stress resistance

In this study, various bioinformatics approaches were used to determine potentially important biomarkers that have functions in Se-dependent oxidative stress resistance. Overall, all the genes that were defined to be important in our study by different analysis methods are summarized in Table. Twenty-seven genes were selected based on within or between cell line comparisons that were associated with either selenium or HBV effect. While most of the genes were previously identified in HCC (17 genes) and oxidative stress (20 genes), HOXD1 and CLYBL were shown to be critical for the first time by this study. 

**Table:** Genes identified by within or between cell line comparisons related with either Se or cell line (HBV) effect. The associations of each gene with oxidative stress and/or HCC in previous studies were indicated. Se: Selenium-deficiency effect, HBV: HBV-integration effect, BCL: Between cell line, WCL: Within cell line, (E): Existing DEG, (S): Steiner node, HM: Heat map, OS: Oxidative stress, r: Reported.

Gene	Effect	Comparison	Analysis	OS	HCC	Literature
FOXA1	Se	BCL	PCST (E)	r	r	Zhang et al. (2005), Song et al. (2009)
CYP7A1	Se	BCL	PCST (E)	r		Liu et al. (2016)
ONECUT1	Se	BCL	PCST (S)		r	Iizuka et al. (2003)
PITX2	Se	BCL	PCST (S)	r	r	Archer et al. (2010), Strungaru et al. (2011)
TXNRD1	Se	BCL	PCST (E)	r	r	Kiermayer (2007), Lee et al. (2019)
ALDH1L2	Se	BCL	PCST (E)	r	r	Lee et al. (2017), Sarret et al. (2019)
ACLY	Se	BCL	PCST (S)	r	r	Migita et al. (2013), Pope et al. (2019)
TXNIP	Se	BCL	PCST (S)	r		Zhou et al. (2013)
SCD5	Se	BCL	PCST (S)	r	r	Yu et al. (2018)
MTR	Se	BCL	PCST (S)	r		Si et al. (2016)
TXNDC17	Se	BCL	PCST (S)	r		Liyanage et al. (2019)
LSM4	Se	BCL	PCST (S)	r		Chen et al. (2017)
CNBP	Se	BCL	PCST (S)	r		de Peralta et al. (2016)
DMPK	Se	BCL	PCST (S)	r		Pantic et al. (2013)
QDPR	Se	BCL	PCST (S)	r	r	Gu et al. (2017), Nwosu et al. (2017)
DUT	Se	WCL	GSEA		r	Takatori (2010)
POLD3	Se	WCL	GSEA	r	r	Jiang et al. (2019), Tan et al. (2020)
E2F2	Se	WCL	GSEA	r	r	Castillo et al. (2015), Huang et al. (2019)
GINS2	Se	WCL	GSEA	r	r	Lian et al. (2018), Liu et al. (2019)
PIK3R3	Se	WCL	GSEA	r	r	Engedal et al. (2018), Ibrahim et al. (2018)
TMEM97	Se	WCL	GSEA	r		Wang et al. (2020)
FGF13	HBV	BCL	HM	r	r	Coleman et al. (2014), Bublik et al. (2017)
GPC3	HBV	BCL	HM		r	Akutsu et al. (2010), Guo et al. (2020)
MAP7D2	HBV	BCL	HM		r	Nishida et al. (2014)
PPAP2A	Se	BCL	HM		r	Jenkins et al. (2012), Nwosu et al. (2017)
HOXD1	Se	BCL	HM			
CLYBL	Se	BCL	HM			

Fifteen DEGs with high betweenness centrality values were identified by the PCST analysis for the between cell line comparison results, and eleven of these genes were Steiner nodes which were not differentially expressed but were determined to be on key positions on pathways that might have effects on the expression of the determined DEGs having indirect effects on differential response. With the GSEA, six DEGs (DUT, POLD3, E2F2, GINS2, PIK3R3, TMEM97) as a result of within cell line comparisons were determined to be key elements for the Se effect according to their highest enrichment scores. Three genes from each HBV and Se effect DEGs were selected from the heat map analysis results with the smallest p-values that have the most significant impact on the differential response to Se- deficiency between the isogenic HepG2 and HepG2-2.2.15 cell lines. 

**Figure 6:**
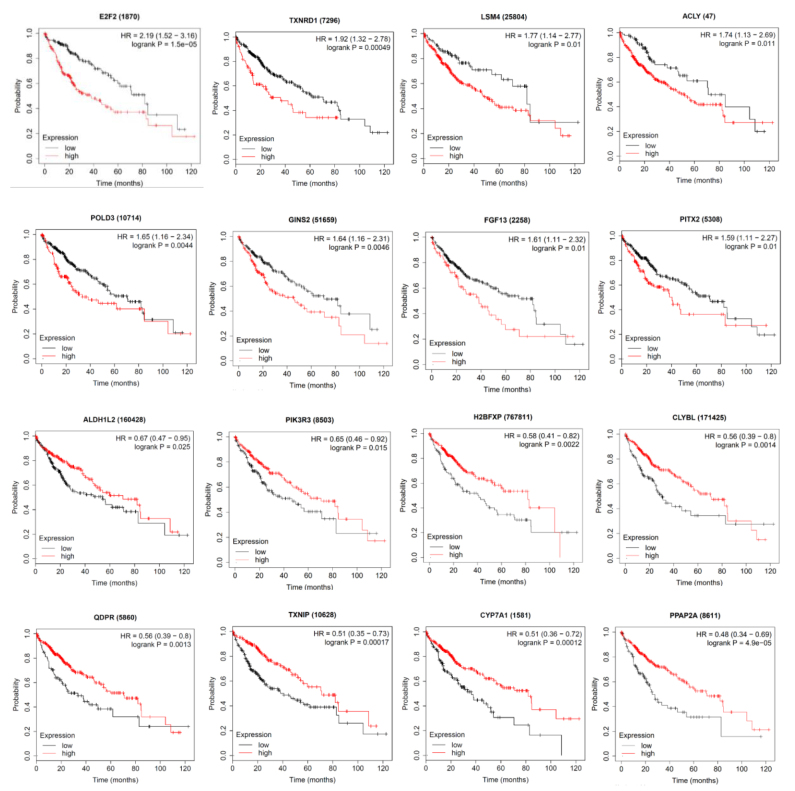
The Kaplan Meier plots were generated for selected DEGs in HCC patients in liver cancer RNA-seq dataset by considering Overall Survival data.

Finally, to find out the clinical relevance of the selected genes; Kaplan–Meier plots were drawn for the liver cancer RNA-seq results (Figure 6). The expression levels of 16 out of 27 genes were found to be associated with overall survival time of the HCC patients.

## 4. Discussion

In this study, the aim was to determine Se-deficiency-dependent oxidative-stress-related gene expression profiles of two isogenic HCC cell lines varying with respect to HBV integration, to identify genes that could be targeted with novel diagnostic and therapeutic strategies. With this aim, the results of an Affymetrix expression array were analyzed and the DEG lists were identified using limma analysis. We found that HBV integration had a larger effect on differential gene expression compared to that of Se-deficiency when the DEG numbers were taken into consideration (Figure 2). The genes thought to play key roles in the differential response to Se-deficiency-dependent oxidative stress were determined by clustering, GSEA as well as network analysis methods, and 27 genes were identified to be the most significant ones and can provide important leads in further studies of HCC diagnosis and therapy. 

Protein–protein interaction (PPI) networks are powerful tools to define some key protein nodes within cell signaling networks. Some values such as degree of a node and betweenness centrality might be used to determine biologically important hub proteins (Yu et al., 2007). The eleven nodes identified by PCST analysis were Steiner nodes and previous studies have shown that such hidden nodes are able to connect the pathways and also indicate cross talks (Huang et al., 2009). Fourteen of the genes identified by PCST were previously shown to have functions in oxidative stress response and seven of those were also known to be related to HCC (Table). Among the six genes identified by GSEA, four (POLD3, E2F2, GINS2, PIK3R3) were previously shown to have oxidative stress and hepatocellular carcinogenesis related functions (Table). Moreover, the association of four of the six DEGs identified by heat map analysis with HCC was shown in previous studies. These results suggest that our statistical methodology has revealed cancer pathways that could be related with hepatocarcinogenesis and extrinsic factors. HOXD1 and CLYBL genes were not associated with any oxidative stress or hepatocellular-carcinogenesis-related function to our knowledge.

GSEA results also indicated that the expression of 15 of those genes was regulated by different combinations of six transcription factors as shown in Table S2, i.e. HSD17B8, CHX10, ZBTB5, TFCP2, LYF1, and E2F2. These transcription factors might be the main targets that should be further investigated for their potential in novel diagnostics and therapeutic strategies. The expression of 16 genes among 27 genes was found to be significantly related to clinical results; the lower expression of ACLY, LSM4, PITX2, TXNRD1, POLD3, GINS2, FGF13, E2F2 and higher expression of ALDH1L2, CYP7A1, QDPR, TXNIIP, PPAP2A, PIK3R3, H2BFXP, CLYBL genes were found to have significant effects on liver cancer patient life spans as shown by the Kaplan–Meier plots (Figure 6). Since various analysis methods were used, it is difficult to make a straightforward association between the survival data and our overall analysis results. However, it is worth exploiting the mechanism of action of these genes/pathways to verify their clinical significance in HCC.

The identification of genes that could be used to predict the prognosis of HCC patients, or lead to the discovery of new therapeutic strategies is important since cancer cells find alternative pathways to compensate the effects of targeted therapies that are currently being used. For this reason, the identification of novel biomarkers that play key roles on those compensatory pathways is very critical. The use of computational methods to analyze the regulation of gene expression values in cancer cells is a powerful guide to develop novel targeted-therapeutic strategies. The outputs of this study emphasize the role of DEGs regardless of Se status and as a result of HBV-integration effect as potential targets in HBV-dependent HCC treatments. Moreover, the effects of selenium-rich diet on the treatment of HCC patients might further be studied to reveal genes identified as potential drivers of Se-deficiency effect in this study. 

Supplementary Materials

## References

[ref1] (2010). Association of glypican-3 expression with growth signaling molecules in hepatocellular carcinoma. World Journal of Gastroenterology.

[ref2] (2010). High-throughput assessment of CpG site methylation for distinguishing between HCV-cirrhosis and HCV-associated hepatocellular carcinoma. Molecular Genetics and Genomics.

[ref3] (2018). GLOBOCAN estimates of incidence and mortality worldwide for 36 cancers in 185 countries. Global cancer statistics.

[ref4] (2017). Regulatory module involving FGF13, miR-504, and p53 regulates ribosomal biogenesis and supports cancer cell survival. Proceedings of the National Academy of Sciences of the United States of America.

[ref5] (2015). E2F1 and E2F2 induction in response to DNA damage preserves genomic stability in neuronal cells. Cell Cycle.

[ref6] (2019). Cryptochrome deletion in p53 mutant mice enhances apoptotic and anti-tumorigenic responses to UV damage at the transcriptome level. Functional & Integrative Genomics.

[ref7] (2020). Harnessing big ‘omics’ data and AI for drug discovery in hepatocellular carcinoma. Nature reviews.

[ref8] (2017). Relationships between stress granules, oxidative stress, and neurodegenerative diseases. Oxidative Medicine and Cellular Longevity.

[ref9] (2014). Fibroblast growth factor family as a potential target in the treatment of hepatocellular carcinoma. Journal of Hepatocellular Carcinoma.

[ref10] (2016). Cnbp ameliorates Treacher Collins Syndrome craniofacial anomalies through a pathway that involves redox-responsive genes. Cell Death and Disease.

[ref11] (2015). Transcriptional up-regulation of APE1/Ref-1 in hepatic tumor: role in hepatocytes resistance to oxidative stress and apoptosis. PLoS One.

[ref12] (2002). Gene Expression Omnibus: NCBI gene expression and hybridization array data repository. Nucleic Acids Research.

[ref13] (2018). From oxidative stress damage to pathways, networks, and autophagy via MicroRNAs. Oxidative Medicine and Cellular Longevity.

[ref14] (2010). Cellular stress responses: cell survival and cell death. International Journal of Cell Biology.

[ref15] (2017). heatmaply: an R package for creating interactive cluster heatmaps for online publishing. Bioinformatics.

[ref16] (2017). Protective effect of dihydropteridine reductase against oxidative stress is abolished with A278C mutation. Journal of Zhejiang University. Science B.

[ref17] (2020). Glypican-3: a new target for diagnosis and treatment of hepatocellular carcinoma. Journal of Cancer.

[ref18] (2007). Oxidative stress and cancer: have we moved forward?. The Biochemical Journal 401 (1).

[ref19] (2009). Integrating proteomic, transcriptional, and interactome data reveals hidden components of signaling and regulatory networks. Science Signaling.

[ref20] (2019). Promising diagnostic and prognostic value of E2Fs in human hepatocellular carcinoma. Cancer Management and Research.

[ref21] (2018). PIK3R3 promotes chemotherapeutic sensitivity of colorectal cancer through PIK3R3/NF-kB/TP pathway. Cancer Biology and Therapy.

[ref22] (2003). Differential gene expression in distinct virologic types of hepatocellular carcinoma: association with liver cirrhosis. Oncogene.

[ref23] (2003). Acquired tolerance of hepatocellular carcinoma cells to selenium deficiency: a selective survival mechanism. Cancer Research.

[ref24] (2009). STRING 8--a global view on proteins and their functional interactions in 630 organisms. Nucleic Acids Research.

[ref25] (2012). Synovial expression of Th17-related and cancer-associated genes is regulated by the arthritis severity locus Cia10. Genes and Immunity.

[ref26] (2019). Microarray-based measurement of microRNA-449c-5p levels in hepatocellular carcinoma and bioinformatic analysis of potential signaling pathways. Pathology Research and Practice.

[ref27] (2007). Effect of selenium on thioredoxin reductase activity in Txnrd1 or Txnrd2 hemizygous mice. Biological Chemistry.

[ref28] (1979).

[ref29] (2019). Induction of oxidative stress through inhibition of thioredoxin reductase 1 is an effective therapeutic approach for hepatocellular carcinoma. Hepatology.

[ref30] (2017). Folate cycle enzyme MTHFD1L confers metabolic advantages in hepatocellular carcinoma. The Journal of Clinical Investigation.

[ref31] (2018). Up-regulated and interrelated expressions of GINS subunits predict poor prognosis in hepatocellular carcinoma. Bioscience Reports.

[ref32] (2019). GINS complex subunit 2 (GINS2) plays a protective role in alcohol-induced brain injury. Artificial Cells, Nanomedicine, and Biotechnology.

[ref33] (2016). Cholesterol 7α-hydroxylase protects the liver from inflammation and fibrosis by maintaining cholesterol homeostasis. Journal of Lipid Research.

[ref34] (2019). Identification of thioredoxin domain-containing protein 17 from big-belly seahorse Hippocampus abdominalis: Molecular insights, immune responses, and functional characterization. Fish & Shellfish Immunology.

[ref35] (2018). Determining consistent prognostic biomarkers of overall survival and vascular invasion in hepatocellular carcinoma. Royal Society Open Science.

[ref36] (2013). Inhibition of ATP citrate lyase induces an anticancer effect via reactive oxygen species: AMPK as a predictive biomarker for therapeutic impact. The American Journal of Pathology.

[ref37] (2017). Identification of the Consistently Altered Metabolic Targets in Human Hepatocellular Carcinoma. Cellular and Molecular Gastroenterology and Hepatology.

[ref38] (2014). Identification of epigenetically inactivated genes in human hepatocellular carcinoma by integrative analyses of methylation profiling and pharmacological unmasking. Digestive Diseases.

[ref39] (2013). Myotonic dystrophy protein kinase (DMPK) prevents ROS-induced cell death by assembling a hexokinase II-Src complex on the mitochondrial surface. Cell Death and Disease.

[ref40] (2007). From selenium to selenoproteins: synthesis, identity, and their role in human health. Antioxidant and Redox Signaling.

[ref41] (2019). Aberrant lipid metabolism as a therapeutic target in liver cancer. Expert Opinion on Therapeutic Targets.

[ref42] (2015). limma powers differential expression analyses for RNA-sequencing and microarray studies. Nucleic Acids Research.

[ref43] (2019). Deleterious mutations in ALDH1L2 suggest a novel cause for neuro-ichthyotic syndrome. NPJ Genomic Medicine.

[ref44] (2020). Cancer Journal for Clinicians.

[ref45] (2016). Overexpression of mycothiol disulfide reductase enhances corynebacterium glutamicum robustness by modulating cellular redox homeostasis and antioxidant proteins under oxidative stress. Scientific Reports.

[ref46] (2009). Role of Foxa1 in regulation of bcl2 expression during oxidative-stress-induced apoptosis in A549 type II pneumocytes. Cell Stress Chaperones.

[ref47] (2011). PITX2 is involved in stress response in cultured human trabecular meshwork cells through regulation of SLC13A3. Investigative ophthalmology and visual science.

[ref48] (2005). Gene set enrichment analysis: a knowledge-based approach for interpreting genome-wide expression profiles. Proceedings of the National Academy of Sciences of the United States of America.

[ref49] (2010). dUTP pyrophosphatase expression correlates with a poor prognosis in hepatocellular carcinoma. Liver International.

[ref50] (2020). An R-loop-initiated CSB-RAD52-POLD3 pathway suppresses ROS-induced telomeric DNA breaks. Nucleic Acids Research.

[ref51] (2016). Network modeling identifies patient-specific pathways in glioblastoma. Scientific Reports.

[ref52] (2020). Functional study of the AMD-associated gene TMEM97 in retinal pigmented epithelium using CRISPR interference. BioRxiv. doi: 10.

[ref53] (2006). Reactive oxygen species: role in the development of cancer and various chronic conditions. Journal of Carcinogenesis.

[ref54] (2007). The importance of bottlenecks in protein networks: correlation with gene essentiality and expression dynamics. PLoS Computational Biology.

[ref55] (2018). Polymorphisms in the 3’-UTR of SCD5 gene are associated with hepatocellular carcinoma in Korean population. Molecular Biology Reports.

[ref56] (1998). Regulation of cell growth by IL-2: role of STAT5 in protection from apoptosis but not in cell cycle progression. The Journal of Immunology.

[ref57] (2005). Microarray data mining for potential selenium targets in chemoprevention of prostate cancer. Cancer Genomics Proteomics.

[ref58] (2013). Roles of thioredoxin binding protein (TXNIP) in oxidative stress, apoptosis, and cancer. Mitochondrion.

