# Supplementary material for: Transcriptome profiles associated with selenium-deficiency-dependent oxidative stress identify potential diagnostic and therapeutic targets in liver cancer cells
Source: Turk J Biol. 2021 Apr 20;45(2):149–61. doi: 10.3906/biy-2009-56 (PMC8068766; doi:10.3906/biy-2009-56)
Supplement: Supplementary file 1 — Supplementary Materials [file turkjbio-45-149-sup001.pdf]

Supplementary Information

Table S1. DEG scores were given for each gene for day 3 according to the indicated formula and (A) the GSEA was performed to find the enriched GO BP_pathways with their enrichment scores. The highlighted results indicate DNA repair and oxidative-stress-related GO terms.

NAME	SIZE	ES	NOM p-val	FDR q-val
CELL_CYCLE	156	0.49	0.00	0.00
DNA_DEPENDENT_DNA_REPLICATION	40	0.64	0.00	0.00
CELL_CYCLE_PHASE_TRANSITION	63	0.57	0.00	0.00
DNA_REPLICATION	51	0.59	0.00	0.00
CELL_CYCLE_PROCESS	125	0.50	0.00	0.00
CELL_CYCLE_G1_S_PHASE_TRANSITION	32	0.65	0.00	0.00
MITOTIC_CELL_CYCLE	96	0.50	0.00	0.00
DNA_METABOLIC_PROCESS	90	0.51	0.00	0.00
CELLULAR_RESPONSE_TO_DNA_DAMAGE_STIMULUS	72	0.50	0.00	0.00
DNA_REPLICATION_INITIATION	17	0.74	0.00	0.00
REGULATION_OF_CELL_CYCLE_PHASE_TRANSITION	41	0.57	0.00	0.00
CELL_CYCLE_DNA_REPLICATION	23	0.63	0.00	0.00
REGULATION_OF_CELL_CYCLE	89	0.45	0.00	0.00
REGULATION_OF_MITOTIC_CELL_CYCLE	52	0.49	0.00	0.00
DNA_REPAIR	56	0.47	0.00	0.00
POSITIVE_REGULATION_OF_MITOTIC_CELL_CYCLE	18	0.62	0.00	0.00
TELOMERE_ORGANIZATION	22	0.59	0.00	0.00
NUCLEAR_DNA_REPLICATION	21	0.60	0.00	0.00
REGULATION_OF_CELL_CYCLE_PROCESS	68	0.45	0.00	0.00
POSITIVE_REGULATION_OF_CELL_CYCLE_PROCESS	29	0.54	0.00	0.00
POSITIVE_REGULATION_OF_CELL_CYCLE_PHASE_TRANSITION	15	0.66	0.00	0.00
POSITIVE_REGULATION_OF_CELL_CYCLE	35	0.50	0.00	0.00
CHROMOSOME_ORGANIZATION	106	0.40	0.00	0.00
REGULATION_OF_DNA_REPLICATION	17	0.60	0.00	0.00
CELL_DIVISION	57	0.43	0.00	0.00
NEGATIVE_REGULATION_OF_CELL_CYCLE	47	0.45	0.00	0.00
ORGANELLE_LOCALIZATION	28	0.51	0.00	0.01
RESPONSE_TO_RADIATION	32	0.50	0.00	0.01
SIGNAL_TRANSDUCTION_BY_P53_CLASS_MEDIATOR	22	0.55	0.00	0.01
CELL_CYCLE_G2_M_PHASE_TRANSITION	25	0.52	0.00	0.01
NEGATIVE_REGULATION_OF_CELL_CYCLE_PHASE_TRANSITION	22	0.54	0.00	0.01
ORGANELLE_FISSION	50	0.43	0.00	0.01
DNA_GEOMETRIC_CHANGE	17	0.57	0.00	0.01
NEGATIVE_REGULATION_OF_CELL_CYCLE_PROCESS	30	0.48	0.00	0.01
ESTABLISHMENT_OF_ORGANELLE_LOCALIZATION	23	0.51	0.00	0.01
DNA_INTEGRITY_CHECKPOINT	18	0.56	0.00	0.01
CHROMOSOME_SEGREGATION	35	0.45	0.00	0.02
REGULATION_OF_CELL_CYCLE_G2_M_PHASE_TRANSITION	21	0.52	0.00	0.02

Table S1. (Continued).

DNA_BIOSYNTHETIC_PROCESS	19	0.53	0.00	0.02
ANATOMICAL_STRUCTURE_HOMEOSTASIS	35	0.45	0.00	0.02
ORGANIC_ACID_METABOLIC_PROCESS	42	0.43	0.00	0.02
REGULATION_OF_SIGNAL_TRANSDUCTION_BY_P53_CLASS_MEDIATOR	15	0.57	0.00	0.02
STEROID_METABOLIC_PROCESS	28	0.48	0.00	0.02
MEIOTIC_CELL_CYCLE_PROCESS	23	0.50	0.00	0.02
DNA_RECOMBINATION	31	0.46	0.01	0.02
MEIOTIC_CELL_CYCLE	28	0.47	0.01	0.02
DOUBLE_STRAND_BREAK_REPAIR	26	0.47	0.00	0.03
RECOMBINATIONAL_REPAIR	17	0.54	0.00	0.03
CELLULAR_RESPONSE_TO_ENDOGENOUS_STIMULUS	81	0.36	0.00	0.03
OXIDATION_REDUCTION_PROCESS	57	0.39	0.00	0.03
DNA_CONFORMATION_CHANGE	35	0.43	0.01	0.03
COFACTOR_METABOLIC_PROCESS	30	0.44	0.01	0.03
ESTABLISHMENT_OF_PROTEIN_LOCALIZATION_TO_ORGANELLE	16	0.55	0.00	0.03
CELL_CYCLE_CHECKPOINT	21	0.48	0.01	0.04
MITOTIC_NUCLEAR_DIVISION	32	0.42	0.01	0.04
COENZYME_METABOLIC_PROCESS	15	0.52	0.01	0.04
RESPONSE_TO_LIGHT_STIMULUS	18	0.49	0.01	0.05
SMALL_MOLECULE_METABOLIC_PROCESS	96	0.33	0.00	0.05
SMALL_MOLECULE_BIOSYNTHETIC_PROCESS	49	0.38	0.00	0.05
CELLULAR_RESPONSE_TO_HORMONE_STIMULUS	32	0.42	0.01	0.06
CELL_CYCLE_ARREST	16	0.50	0.01	0.06
PROTEIN_DNA_COMPLEX_SUBUNIT_ORGANIZATION	22	0.46	0.01	0.06
NUCLEAR_CHROMOSOME_SEGREGATION	28	0.42	0.02	0.06
CELLULAR_PROCESS_INVOLVED_IN_REPRODUCTION_IN_MULTICELLULAR_ORGANISM	19	0.49	0.02	0.06
REGULATION_OF_DNA_METABOLIC_PROCESS	19	0.48	0.01	0.07

Table S2. The common transcription factors identified by GSEA analysis and associated with the regulation of genes of interest were marked by circles.

GENE NAME	HSD17B8	CHX10	ZBTB5	TFCP2	LYF1	E2F2
FOXA1	o		o	o		
CYP7A1						
ONECUT1						
PITX2		o		o	o	o
TXNRD1						
ALDH1L2						
ACLY						
TXNIP			o			
SCD5						
MTR			o			o
TXNDC17				o		
LSM4						
CNBP						
DMPK					o	
QDPR						
DUT	o					o
POLD3	o					o
E2F2	o					o
GINS2	o					
PIK3R3		o	o			o
TMEM97	o					o
FGF13			o		o	
GPC3		o		o	o	
MAP7D2						
PPAP2A		o				
HOXD1						
CLYBL						
